# TMAO at the Crossroads of the Gut–Brain Axis: Emerging Mechanisms Linking Microbiota-Derived Metabolites to Neuroinflammation and Cognitive Dysfunction

**DOI:** 10.3390/cells15201845

**Published:** 2026-10-12

**Authors:** Aseel Y. Altahrawi, Fatimah Sofi, Zara Kanjwal, Zahoor A. Shah

**Affiliations:** 1 Department of Pharmacology and Experimental Therapeutics, College of Pharmacy and Pharmaceutical Sciences, The University of Toledo, Toledo, OH 43614, USA; aseel.altahrawi@rockets.utoledo.edu; 2 Department of Medicinal and Biological Chemistry, College of Pharmacy and Pharmaceutical Sciences, The University of Toledo, Toledo, OH 43614, USA

**Keywords:** TMAO, neurological diseases, gut–brain axis, therapeutic strategies

## Abstract

**Highlights:**

**What are the main findings?**

- TMAO is increasingly recognized as a potential target linking gut microbial metabolism with neurovascular dysfunction and neurodegenerative diseases.
- Experimental and clinical evidence suggests that TMAO may affect neurological health through combined mechanisms involving oxidative stress, neuroinflammation, endothelial dysfunction, blood–brain barrier integrity, and altered gut–brain axis signaling.

**What are the implications of the main findings?**

- Understanding how TMAO and other microbial metabolites affect neurovascular, inflammatory, and cellular signaling pathways could identify underrecognized mechanisms linking gut microbial mechanisms with neurological dysfunction.
- Modulation of TMAO synthesis and metabolism may provide a new therapeutic strategy to mitigate or slow the progression of neurological and cerebrovascular diseases.

**Abstract:**

Trimethylamine-N-oxide (TMAO) is a gut microbiota-derived metabolite produced through the hepatic oxidation of trimethylamine. Although TMAO has been widely investigated as a therapeutic target in multiple cardiovascular and metabolic diseases, growing evidence suggests it may play a critical role in the neuroinflammation and cognitive dysfunction associated with the gut–brain axis. This narrative review summarizes the current literature to synthesize evidence on the mechanisms linking TMAO with neurological disorders; it discusses potential mechanisms contributing to this association, with particular focus on neuroinflammation, oxidative stress, and activation of inflammatory pathways. Overall, this review also suggests the potential use of TMAO and its precursors as therapeutic targets for multiple neurodegenerative disorders. However, it emphasizes the need for conducting large, adequately powered randomized clinical studies to determine its therapeutic relevance.

## 1. Introduction

The gut microbiota is a diverse and complex microbial ecosystem in humans and animals. The vital role of gut microbiota symbiosis in regulating human metabolism and the physiological function of different organ systems has recently been a focus of research. Several studies have shown evidence that dysbiosis in the gut microbiota plays a vital role in the etiology of different illnesses. In addition to neural, immune, and hormonal pathways, these effects are mediated by the production of certain metabolites from the gut microbiota [1]. The role of gut microbiota in the development of the central nervous system and in several neurological diseases has been studied extensively over the past decade [2]. Animal studies have further shown that changes in the composition of microbiota lead to neurobehavioral changes in rats [3]. Trimethylamine N-oxide (TMAO) is one of the potential metabolites generated by gut microbiomes. TMAO is generated when gut bacteria metabolize nutrients such as choline, carnitine, and betaine to trimethylamine, which is subsequently converted to TMAO in the liver. Although TMAO has been widely studied in cardiovascular and metabolic diseases, growing evidence suggests that it may also contribute to neurological disorders. However, data linking TMAO to neurological diseases remain limited across different diseases and biological pathways. Previous reviews have discussed TMAO, the gut–brain axis, and gut-derived metabolites’ effects on multiple neurodegenerative diseases; however, a comprehensive understanding of how TMAO may contribute to neurological dysfunction across different disease states is still needed. Therefore, in this narrative review, we discuss evidence from neurodegenerative, cerebrovascular, traumatic, and psychiatric disorders, focusing on neuroinflammation, oxidative stress, blood–brain barrier (BBB) dysfunction, immunological responses, and neuronal injury. By evaluating these pathways across multiple neurological conditions, we aim to clarify the potential role of TMAO within the gut–brain axis and identify common and disease-specific mechanisms. We will also discuss the possibilities of targeting the gut microbiota–TMAO pathway as a possible approach to reduce or prevent neurological dysfunction. Relevant studies were identified through searches of PubMed and Google Scholar using combinations of the terms “trimethylamine-N-oxide (TMAO)”, “Gut-brain axis”, “neuroinflammation”, “cognitive dysfunction”, “oxidative stress”, and “microbiota”. The search included publications from 2010 to 2026. In PubMed, Boolean operators (AND/OR) were used to combine search terms; the primary search strategy was (“trimethylamine N-oxide” OR “TMAO”) AND (“gut–brain axis” OR microbiota OR microbiome) AND (neuroinflammation OR “oxidative stress” OR “cognitive dysfunction” OR cognition). Relevant studies were identified by screening the title and abstracts, followed by full-text review when appropriate. Peer-reviewed original research articles, clinical studies, animal studies, or in vitro studies that investigated TMAO, TMAO-related metabolism, gut microbiota, the gut–brain axis, neuroinflammation, oxidative stress, or cognitive/neurological outcomes were considered. Review articles, systematic reviews, and meta-analyses were also considered for background information and identification of additional relevant primary studies. Studies were excluded when they were unrelated to the scope of the review, lacked sufficient information regarding TMAO or the relevant neurological/gut–brain mechanisms, or were duplicate publications. As this was a narrative review, no formal risk of bias assessment or predefined systematic-review framework was applied.

### 1.1. TMAO Metabolism

The microbial action on dietary choline and phosphatidylcholine in the gut leads to the production of trimethylamine (TMA), which is absorbed into the bloodstream and is oxidized to TMAO by Flavin oxygenase (FMO-1 and-3) in the liver [4]. TMAO is mostly cleared by the kidneys, and small amounts are excreted in feces, sweat, or exhaled air. However, a small portion of TMAO is transported to the tissues for accumulation as an osmolyte to maintain cell volume [5]. Certain bacterial species, which include *Firmicutes* and *Proteobacteria*, *Anaerococcus hydrogenalis*, *Clostridium asparagiforme*, *C. hathewayi*, *C. sporogenes*, *Escherichia fergusonii*, *Proteus penneri*, *Providencia rettgeri*, and *Edwardsiella tarda*, have been associated with TMA/TMAO production [6]. In addition to the gut microbial flora, TMAO levels may also depend on dietary factors and renal function [6]. Consumption of a diet with eggs, beef, and fish has been shown to increase the levels of TMA/TMAO in both blood and urine [7]. However, a few other studies failed to show any association with the diet [8]. Serum TMAO levels have a strong association with renal function with higher levels in chronic kidney disease [9]. Furthermore, TMA/TMAO levels have been shown to decrease significantly after kidney transplant [10].

Elevated TMAO levels have been associated with the development of cardiovascular diseases, chronic kidney disease and new evidence also suggests a link with pulmonary diseases [11,12,13]. In the vascular system, TMAO may contribute to endothelial dysfunction by activating inflammatory and oxidative stress pathways. Experimental studies have shown that TMAO activates protein kinase C (PKC) and NF-κB signaling in endothelial cells, leading to increased expression of vascular adhesion-1 (VCAM-1) and improved monocyte adhesion [14]. TMAO has also been associated with reduced nitric oxide (NO) signaling, including reduced endothelial nitric oxide synthase (eNOS) activity, decreased NO bioavailability, and increased production of reactive oxygen species (ROS), which further contribute to endothelial dysfunction [15]. These changes may precipitate vascular inflammation, facilitating the recruitment and retention of circulating monocytes within the vascular wall. Once recruited to the vascular wall, monocytes can differentiate into macrophages, leading to lipid accumulation and foam cell formation, ultimately contributing to the development of atherosclerotic lesions [16].

### 1.2. Physiological Roles of TMAO

Consuming animal-based foods, such as red meat, eggs, or fish, provides several nutrients for the body, including L-carnitine, choline, and betaine. Bacteria in the gut known as Firmicutes feed on these nutrients and convert them into a gas called TMA, which is then absorbed into the circulation and transported to the liver. Hepatic flavin-containing monooxygenase-3 (FMO3) is the major enzyme responsible for oxidizing TMA to trimethylamine N-oxide (TMAO. Notably, TMAO concentrations are influenced by multiple factors, such as dietary intake, gut microbial metabolism, as well as the regulation of FMO3. FMO3 expression can be regulated by hormonal and metabolic signals, including androgens and bile acid-activated farnesoid X receptor (FXR) signaling. Previous studies have demonstrated that increased hepatic FMO3 expression increased circulating TMAO levels, whereas suppressing FMO3 was associated with decreased TMAO production [17,18].

**Figure 1:**
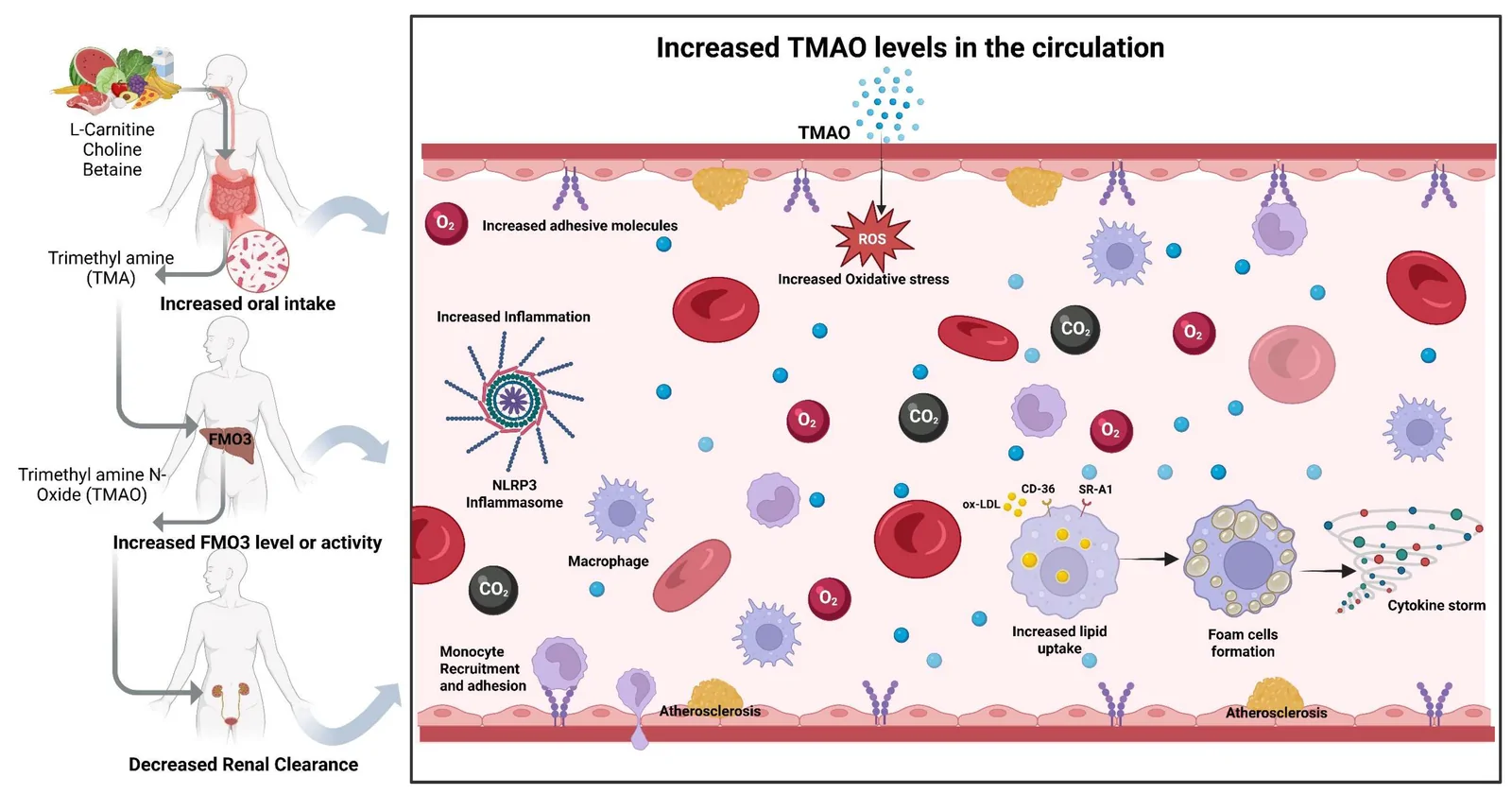
Proposed inflammatory mechanisms underlying TMAO-induced atherosclerotic lesions. Gut microorganisms convert dietary precursors, such as choline, L-carnitine, and betaine, to trimethylamine (TMA), which is then transformed to TMAO in the liver by FMO3. Increased dietary precursor availability, higher FMO3 expression or activity, and decreased renal clearance can all contribute to increased circulating TMAO levels. Elevated TMAO levels can lead to endothelial dysfunction, oxidative stress, and inflammatory signaling through pathways such as the NLRP3 inflammasome and NF-κB, resulting in increased monocyte recruitment and vascular inflammation. In macrophages, TMAO increased CD36/SR-A1 mediated ox-LDL uptake and lipid accumulation, leading to foam cell formation and atherosclerotic plaque growth. FMO, flavin monooxygenase; TMA, trimethylamine; TMAO, trimethylamine N-oxide.

After TMAO formation, it is eliminated primarily through the kidneys. Renal clearance includes active tubular transport and active filtration. Organic cation transporter 2 (OCT2) has been identified as an important transporter involved in TMAO uptake into renal tubular cells, while multidrug and toxin extrusion protein 1 (MATE1) supports its movement across the apical membrane and its renal secretion. Therefore, any change in renal function or transporter activity can reduce TMAO clearance and enhance its accumulation in the systemic circulation [19,20]. Thus, circulating TMAO levels reflect a combined effect of dietary precursor availability, gut microbial TMA production, hepatic FMO3 activity, and renal elimination. Increased TMAO levels have been associated with vascular inflammation and atherosclerotic disease, although the contribution of these metabolic determinants may differ among individuals. Figure 1 summarizes the potential mechanisms linking gut microbiota-derived TMAO to the development of atherosclerosis.

#### 1.2.1. Foam Cell Formation

Foam cell formation is an important phase in the development of atherosclerotic plaques. It happens when macrophages accumulate an excessive amount of modified lipids, specifically oxidized low-density lipoprotein (Ox-LDL). Under normal physiological conditions, macrophages maintain cholesterol homeostasis by balancing lipid uptake, intracellular processing, and cholesterol efflux. However, in an atherosclerotic environment, increased uptake of ox-LDL through multiple receptors such as CD36 and scavenger receptor class A1 (SR-A1), together with impaired cholesterol excretion through transporters such as ABCA1 and ABCG1, will promote intracellular lipid accumulation and the conversion of macrophages into foam cells [21].

Recent studies suggest that TMAO can directly affect these processes rather than being associated with increased risk for atherosclerotic diseases. Several studies have shown that TMAO increased CD36 and SR-A1 expression in macrophages, enhanced ox-LDL absorption, and foam cell formation. In ApoE-deficient mice, TMAO injection improved macrophage recruitment and CD36 expression within the atherosclerotic lesion; similarly, in cultured macrophages, TMAO enhanced ox-LDL-induced lipid accumulation [22]. Interestingly, inhibition of CD36 or pharmacological blocking of MAPK/JNK signaling reduced TMAO-induced CD36 expression and foam cell formation, which supports the role of the CD36-MAPK/JNK pathway in this process [22]. TMAO has also been reported to interfere with cholesterol excretion mechanisms such as ABCA1 and ABCG1, which further promotes lipid retention inside macrophages [23]. These findings suggest that TMAO may promote foam cell formation through a combination of increased lipid uptake, disrupted cholesterol, and activation of inflammatory signaling.

TMAO may additionally promote macrophage recruitment and inflammatory activation in the developing plaque. In an experimental study, TMAO increased macrophage migration and the expression of proinflammatory mediators such as TNF-α, IL-6, and ICAM-1, which were associated with activation of MAPK/JNK signaling [22]. Overall, increased monocyte and macrophage recruitment, enhanced lipid uptake and impaired cholesterol modification can promote foam cell formation and atherosclerotic lesion progression.

#### 1.2.2. Endothelial Dysfunction

An early event that connects vascular stress to the development of atherosclerotic lesions is endothelial dysfunction. Endothelial cells activate transcriptional pathways like NF-κB and mitogen-activated protein kinases (MAPKs), including p38, ERK, and JNK, in response to oxidized lipids, inflammatory mediators, and oxidative stress. Activation of these pathways increases the expression of adhesion molecules and chemokines, including VCAM-1, ICAM-1, E-selectin, and monocyte chemoattractant protein-1 (MCP-1), which promote leukocyte adhesion and recruitment to the vascular wall [23,24]. This could be an important molecular link between endothelial activation and monocyte infiltration, macrophage accumulation, and plaque development [25].

In addition, TMAO has been demonstrated to increase ROS levels and activate the ROS–thioredoxin interacting protein (YXNIP)–NLRP3 inflammasome pathway. The activation of this pathway promotes the production of multiple inflammatory mediators such as IL-1β and IL-18, while decreasing eNOS activity and NO availability [26]. Since NO is required for vascular dilation and endothelial homeostasis, its reduction can exacerbate endothelial dysfunction. Moreover, TMAO has been linked to decreased endothelial repair capacity by affecting the endothelial progenitor cells [23]. These endothelial changes can precipitate the recruitment of additional monocytes into the arterial wall, where they differentiate into macrophages and participate in TMAO-induced foam cell formation.

#### 1.2.3. Plaque Instability

Atherosclerotic plaques are composed of a lipid-rich core and a collagen-rich fibrous covering, referred to as the “fibrous cap”. As the lipid-rich core accumulates, the covering component weakens, enhancing inflammation and leaving the plaque vulnerable to rupture. High TMAO levels actively drive plaques into these dangerous, vulnerable states by increasing the volume of the lipid-rich core and thinning the fibrous cap, making the vessel much more fragile. This leaves patients at risk of a heart attack or stroke [25].

### 1.3. TMAO and Association with Alzheimer’s Disease (AD)

Neuroinflammation has been linked to normal cognitive aging [27]. This is mediated by astrocyte activation and higher levels of proinflammatory cytokines [28]. Different pathophysiological cascades, including insulin resistance, mitochondrial dysfunction, or cerebral hypoperfusion, have been postulated to explain neurodegenerative changes [29]. This is explained in a little more detail below. Additionally, dysbiosis in normal aging has also been postulated as a possible mechanism for cognitive decline. Dysbiosis can reduce the expression of tight junction proteins in the gut, which in turn will lead to intestinal inflammation. Intestinal inflammation increases intestinal mucosal permeability, favoring the passage of metabolites produced in the gut [30]. TMAO is one of the potential proinflammatory metabolites produced from the gut lumen, which may cross the gut epithelium into the bloodstream. Several studies have shown the systemic proinflammatory activity of TMAO [31]. Proinflammatory activity can compromise the blood–brain barrier (BBB), which could increase permeability to neurotoxic metabolites [32,33]. Neurotoxic metabolites in the brain can cause neural injury, leading to neuroinflammation [34]. Amyloid plaque deposition is associated with microglial-mediated neuroinflammation [35]. Several studies have shown an association of TMAO with increased aggregation of intrinsically disordered proteins (IDPs), including Aβ42, which is involved in the pathogenesis of Alzheimer’s disease [36,37]. Higher levels of TMAO were found in cerebral spinal fluid (CSF) of patients with AD. In addition, CSF TMAO was associated with p-tau as well as p-tau/Aβ42 [38]. However, another study found no causal relationship between TMAO and AD [39]. Therefore, more studies are needed to clearly establish TMAO’s role, if any, in neurodegenerative disorders.

The mechanism of TMAO-induced neurodegeneration could be multifactorial. Animal studies have shown the role of gut microbiota and TMAO in platelet hyperresponsiveness and thrombosis [40]. Gut microbiota-induced elevation of TMAO has been shown to accelerate atherosclerosis in mice [4,41]. Atherosclerosis is considered an independent risk factor for dementia [42]. TMAO could potentially induce vascular inflammation by activating the NLRP_3_ inflammasome [43]. TMAO has been shown to increase synaptic damage and reduce the expression levels of synaptic plasticity-related proteins in mice. This is mediated by inhibiting the mTOR signaling pathway, which aggravates aging-related cognitive dysfunction. TMAO treatment in mice was shown to reduce superoxide dismutase (SDM) and Glutathione peroxidase (GPX) activity, leading to oxidative stress. Additionally, SOD activity was reduced, and H_2_O_2_ activity was increased in the hippocampus in these specimens [44]. TMAO could potentially promote vascular inflammation by activating the nuclear factor-κB signaling cascade, which induces inflammatory gene expression in endothelial cells and vascular smooth muscle and promotes leukocyte adhesion to endothelial cells [45]. TMAO has also been shown to exacerbate impaired glucose tolerance and increase fasting insulin levels, which is associated with insulin resistance [46]. Insulin resistance leads to hyperinsulinemia, which may have a role in pathological and functional abnormalities seen in Alzheimer’s disease [47]. TMAO treatment has also been shown to impact the expression of certain proteins in 5XFAD mice, including proteins that affect neuronal health and amyloid-β precursor protein processing in the brain. This results in reduced neurite density in several brain regions [48]. In ex vivo models, impaired synaptic transmission was observed when wild-type hippocampal brain slices were incubated with TMAO. Additionally, the endoplasmic reticulum (PERK-EIF2α-ER) stress signaling axis was induced in TMAO-treated hippocampal slices. Altered presynaptic and postsynaptic receptor expression was also noted in these specimens treated with TMAO [49]. Some studies failed to show any association between TMAO and neurological disorders. In a large population-based study of 3143 subjects, TMAO showed no association with cognition, neuroimaging markers, or incident dementia. On the other hand, higher levels of plasma choline, a precursor of TMAO, were associated with reduced cognition, brain atrophy, and cerebral small vessel disease [50]. Another longitudinal study involving 529 subjects and a 13-year follow-up found no association between TMAO and cognitive impairment in multivariate analysis. On the other hand, higher plasma levels of crotonobetaine were associated with a significant risk of cognitive impairment and dementia [51].

### 1.4. TMAO and Association with Parkinson’s Disease (PD)

Loss of neurons in the substantia nigra is the hallmark of PD. This is associated with the accumulation of ubiquitinated α-synuclein and other proteins in cytoplasmic inclusions. TMAO induces folding of the α-synuclein peptide, which can lead to fibrillation and aggregation or formation of monomers [52]. Accumulation of misfolded α-synuclein leads to impairment in the functioning of lysosomes and mitochondria, as well as damage to the cytoskeleton and accumulation of Lewy Body inclusions [53]. In MPTP-induced PD mice, elevated TMAO levels were associated with worsening glial reaction and neuroinflammation, leading to increased dopaminergic neural loss [54]. Additionally, higher TMAO levels impair myelin and oligodendrocyte integrity, which leads to demyelination and accelerated neurodegeneration in PD [55]. Ex vivo studies showed that TMAO-treated midbrain organoids induced expression of senescence markers (P21, P16) and p53 accumulation. In addition, it also showed neurodegeneration phenotypes, including loss of dopaminergic neurons. Furthermore, these midbrain organoids treated with TMAO also showed astrocyte activation and neuromelanin accumulation [56].

On the other hand, human studies showed mixed results. Elevated TMAO levels were demonstrated in CSF and plasma in PD patients [57]. In their exploratory Mendelian randomization study, Zhou et al. examined the causal relationship between TMAO and PD. Suggestive evidence was seen of an inverse causal effect of TMAO on PD progression [58]. However, another study failed to show any evidence of a causal relationship between circulating TMAO and the risk of PD [59].

### 1.5. TMAO Association with Ischemic and Hemorrhagic Strokes

Dysbiosis has been observed following a stroke or transient ischemic attack. Half the patients report GI symptoms following a stroke [60]. Several observational studies have shown an increase in opportunistic pathogens (*Megaspheara*, *Enterobacter*, *Oscillibacter*) and a paucity of beneficial flora, including *Bacteroides*, *Prevotella*, and *Faecalibacterium* [61,62]. Rapid dysbiosis with overgrowth of *Enterobacteriaceae* following ischemic stroke was observed in animal studies, which was shown to worsen brain infarction. In contrast, suppression of Enterobacteriaceae overgrowth with superoxide dismutase or aminoguanidine alleviated brain infarction [63]. Another prospective study found an increase in TMAO-producing genera and a lower abundance of butyrate-producing bacteria [64].

In addition to its role in atherogenesis, as described above, TMAO also promoted platelet adhesion at the damaged endothelium in atherosclerotic arteries, leading to platelet activation and thrombus formation. In animal studies, after intraperitoneal injection of TMAO in mice, thrombosis potential was assessed using a carotid artery injury (FeCl3) model with fluorescently tagged platelets, showing a significantly increased rate of fluorescent thrombus formation (*p* < 0.001). In addition, platelet aggregation in the presence of physiological levels of TMAO led to an initial several-fold increase in intraplatelet calcium levels. Additionally, TMAO augmented inositol-1,4,5-trisphosphate (IP3) signaling pathways in platelets with either submaximal thrombin or ADP [40]. Another study showed that TMAO upregulated vascular cell adhesion molecule-1 (VCAM-1). This TMAO-mediated vascular tissue factor (TF) expression and thrombogenicity was blocked by a TF-inhibitory antibody or gut microbial choline TMA-lyase [65]. An experimental drug, 3,3-dimethyl-1-butanol (DMB), which inhibits microbial choline TMA-lyase, was shown to significantly reduce TMAO and ADP-dependent platelet aggregation in platelet-rich plasma. This protective effect was reversed by direct injection of TMAO [66].

The bidirectional crosstalk between the GI tract and the brain, the so-called gut–brain axis (GBA), is mediated by complex, incompletely understood pathways and affects motor, sensory, and secretory functions of the GIT (top-to-bottom pathway), as well as neurobehavioral functions of the brain (bottom-to-top pathway) [67,68]. The two-way communication in GBA may be mediated through the vagus nerve, spinal cord, and immune pathways involving cytokines and TMAO [69]. In the top-to-bottom pathway, the altered signals transmitted via GBA in ischemic stroke led to defective intestinal barriers and translocation of intestinal bacteria into circulation [70]. Gut dysbiosis following stroke could cause intestinal inflammation and an increased immune response, which could disrupt blood–brain barrier (BBB) function. Increased BBB permeability allows peripheral immune cells and proinflammatory molecules, such as the NLRP3 inflammasome and TMAO, to infiltrate the brain, augmenting the inflammatory cascade at the site of brain ischemia [71]. Patients with disruption of the BBB were associated with larger lesion volumes and more severe strokes [72].

Several animal and clinical studies have shown the association of elevated levels of TMAO with the development of ischemic stroke as well as negative outcomes following a stroke. TMAO has been reported to promote vascular inflammation through activation of nuclear factor-κB [45], while an experimental study in mice has shown increased platelet reactivity and thrombotic potential in the presence of TMAO [40]. These results provide evidence for a potential role of TMAO in pathways related to thrombosis; however, they do not establish that TMAO directly causes ischemic stroke. Several clinical studies show an increase in the levels of TMAO in patients with ischemic stroke on admission [73]. In addition, higher levels of TMAO on admission were found to be associated with more severe stroke intensity [73]. Furthermore, a significant reduction in TMAO levels was observed 48 h after an ischemic stroke [73]. It was hypothesized that TMAO activates thrombosis leading to stroke, and that the drop in TMAO levels after admission was due to alterations in the gut microbiota following ischemic stroke [61]. A prospective study to investigate the correlation of TMAO levels following carotid stenting in patients with carotid stenosis found higher TMAO levels in patients who developed new lesions on MRI following stenting compared to those without new lesions [74].

Arterial stiffening with aging correlated with TMAO levels in a study by Brunt et al. [75]. Markedly elevated TMAO levels were observed in middle-aged and older individuals (6.3 ± 5.8 µmol/L) compared to young subjects (1.8 ± 1.4 µmol/L) (*p* < 0.001), and it showed a positive correlation with aortic pulse wave velocity (PWV) and systolic BP. Similar results were seen in animal experiments in this study. Additionally, dietary supplementation with TMAO increased PWV in young mice and worsened PWV in old mice. They also observed higher levels of advanced glycation end-products (AGE) in the aortic wall of TMAO-treated mice compared with controls. Stiffness in aortic rings increased on ex vivo incubation with TMAO, but this was attenuated with the addition of the experimental drug Alagebrium (ALT-711), which breaks down AGE. However, as TMAO metabolism and vascular aging may differ between mice and humans, it would be difficult to directly translate these animal findings to human vascular aging. Therefore, further clinical and longitudinal studies are needed to clarify whether TMAO has a critical role in vascular stiffening in humans.

TMAO may have a role to play in post-ischemic stroke neurological recovery. In a study by Su et al., neurological function recovery was monitored in rats following middle cerebral artery occlusion/reperfusion (MCAO/R). The expression of Smurf2 and ALK5, which play a crucial role in neuronal differentiation following stroke, was assessed. TMAO was shown to promote reactive astrocyte proliferation and formation of a glial scar by inhibiting the ubiquitination role of Smurf2 and upregulating ALK5 in MCAO/R rats. Furthermore, Smurf2 overexpression and ALK5 silencing abolished this effect [76].

Although hemorrhagic stroke, intracerebral hemorrhagic stroke (ICH), is less frequent than ischemic stroke, it is considered a more severe type of stroke, mainly due to its high mortality and disability burden [77]. As of 2021, ICH accounted for approximately 28.8% of stroke cases worldwide, with more than 3.4 million new cases and 3.3 million deaths, representing 45.6% of all stroke-related deaths [77]. Given the high mortality rate associated with ICH, recent research is focusing on identifying molecular and metabolic factors that may influence ICH progression and outcomes, including the gut microbiota-derived metabolite TMAO. For example, a prospective observational study evaluated 307 patients with acute ICH who presented to the emergency department within 6 h of symptom onset and had their TMAO concentration measured within 24 h of admission. Three months after stroke onset, 139 patients (45.3%) had a poor functional result. After adjusting for potential covariates, patients in the highest quartile of plasma TMAO had significantly higher odds of poor functional outcomes than those in the lowest quartile (adjusted odds ratio (OR) = 3.65, *p* < 0.01), with a significant linear relationship between TMAO level and poor outcomes (*p* = 0.013). Nevertheless, TMAO levels were not significantly associated with early neurological deterioration or hematoma enlargement (*p* > 0.05) [78].

A more recent prospective, multicenter observational cohort study conducted by Sun et al. investigated the association between TMAO and neurological outcomes in patients with severe spontaneous ICH who underwent surgical treatment, in which 40 patients were evaluated using their metabolic profiles, which identified TMAO as one of the metabolites that change consistently between patients with good and poor outcomes at 3, 7, and 30 days post-surgery. These results were further validated with a subsequent prospective cohort study of 794 patients, of whom 147 patients had poor functional outcomes 3 months after surgery. Higher levels of TMAO on day 3 after surgery were significantly associated with 3-month poor functional outcomes (OR = 4.7, *p* < 0.001) [79]. Collectively, these findings suggest that an early postoperative increase in serum TMAO may serve as a biomarker for poor long-term functional outcomes after severe ICH, although the observational design prevents establishing a causal relationship.

Consistent with these clinical observations, experimental evidence further supports TMAO’s pathological role in ICH. Ye et al. investigated the effect of TMAO using a collagenase-induced rat model of ICH. They observed that TMAO administration (900 mg/kg/day for 7 days) significantly exacerbated neurological deficit compared with ICH alone (*p* < 0.01). TMAO administration also significantly decreased locomotor activity and increased microglial activation and IL-1β expression [80]. In vitro experiments showed that TMAO increased ROS production and activated the NLRP3/caspase-1 inflammasome pathway in BV2 microglial cells. Moreover, depletion of the gut microbiota reduced TMAO levels and attenuated L-carnitine-induced activation of Iba-1, NLRP3, caspase-1, and IL-1β. These findings suggest that TMAO could contribute to ICH-induced neuroinflammation via gut microbiota-dependent activation of the NLRP3 inflammasome. These clinical and experimental observations increased the possibility that TMAO may be involved in the pathophysiology of ICH-induced poor functional and neurological outcomes rather than functioning as a marker of disease severity. However, the studies were observational and had relatively small sample sizes, which may allow residual confounding and limited generalizability. Therefore, larger, well-controlled clinical studies are needed to validate the association and to clarify whether TMAO contributes directly to poor outcomes or mainly reflects metabolic changes that occur after ICH.

### 1.6. TMAO and Association with Other Neurodegenerative Diseases

The potential role of TMAO in neurological disease has extended beyond the major neurodegenerative diseases. Recent studies established an association between gut microbiota-derived metabolites and epilepsy, amyotrophic lateral sclerosis (ALS), and other neuropsychiatric disorders. For instance, Ouyang et al. conducted a bidirectional Mendelian randomization study linking the gut microbiota, gut metabolites, and various epilepsy subtypes. They found an association between different microbial species and epilepsy susceptibility, including an increased risk of childhood epilepsy associated with Veillonellaceae (odds ratio (OR): 1.033, *p* = 0.0003). Nevertheless, their analysis of TMAO and its precursor indicated no significant causal association with epilepsy. Thus, further research is warranted to evaluate the effects of TMAO on epilepsy [81]. Likewise, Du et al. evaluated the gut microbiota–brain axis in a rat model of traumatic brain injury (TBI) [82]. They observed a significant increase in both serum and ipsilateral brain TMAO levels. However, fecal microbiota transplantation from healthy rat donors significantly decreased TMAO levels (*p* < 0.05), with TMAO levels approximately 4-fold and brain TMAO levels almost 14-fold higher than in the sham group. FMT levels also increased hippocampal methionine sulfoxide reductase A (MsrA) and improved the expression of antioxidant markers (e.g., SOD, CAT, and reduced glutathione (GSH)) and significantly reduced the production of ROS and MDA (*p* < 0.05). These results suggest that modulation of gut microbiota and reducing TMAO levels may contribute to neurological recovery after TBI, mainly through the TMA-TMAO-MsrA signaling pathway.

Similarly, studies on neuropsychiatric diseases illustrate a detrimental effect. Baranyi et al. observed that people who developed post-traumatic stress disorder (PTSD) post-myocardial infarction (MI) had significantly higher levels of circulating TMAO than those without PTSD symptoms (*F* = 4.544, *p* = 0.035) [83]. Similarly, Meinitzer et al. investigated the association between serum TMAO and the severity of depressive symptoms in patients with or without carbohydrate malabsorption and found a significant positive correlation between TMAO levels and depressive symptom severity (*p* = 0.011, ρ = 0.160). Interestingly, this association was particularly prominent in females (*p* = 0.010, ρ = 0.274) and males (*p* = 0.027, ρ = 0.315) without carbohydrate malabsorption, whereas no significant association was observed in individuals with carbohydrate malabsorption [84].

**Figure 2:**
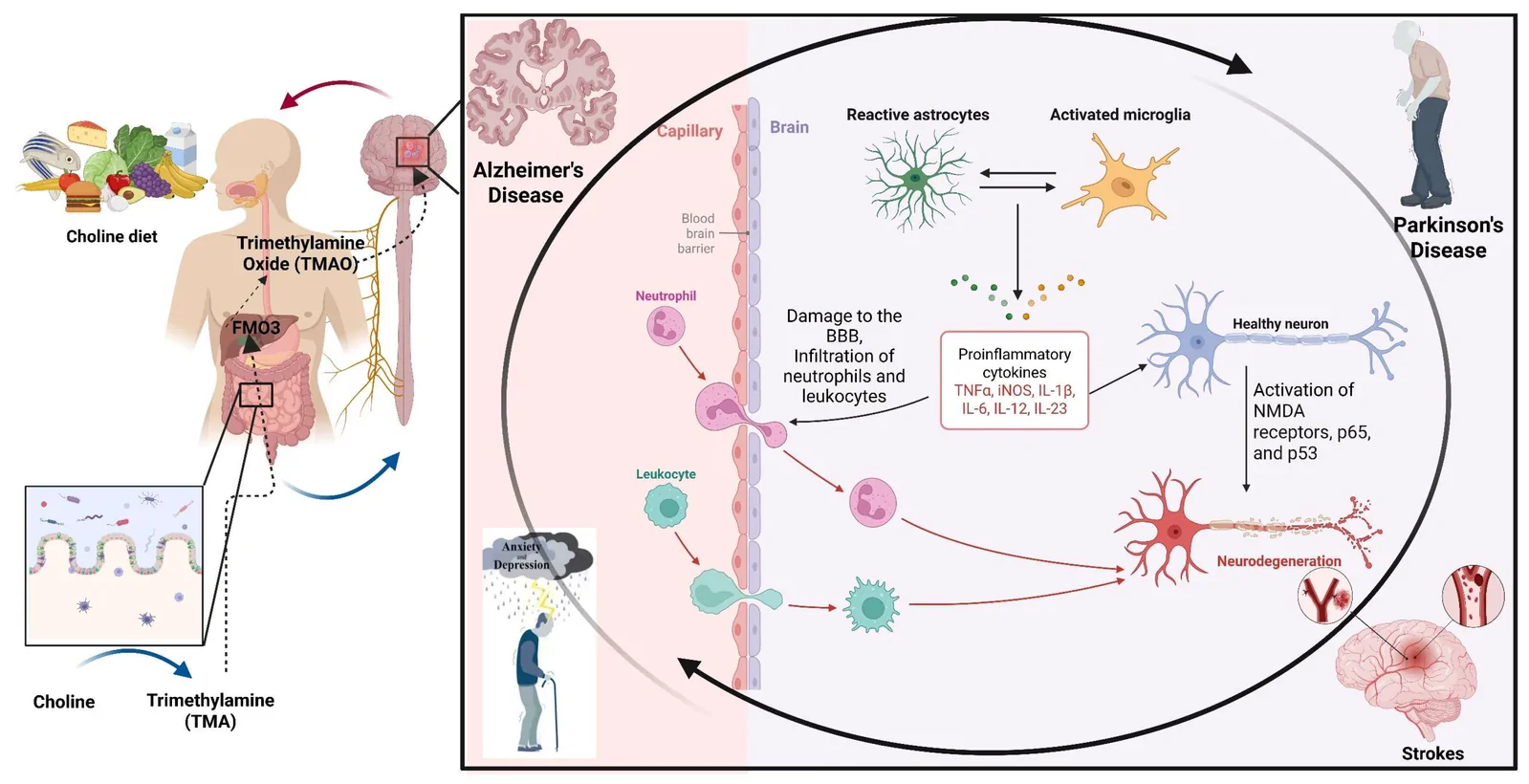
Proposed inflammatory mechanisms underlying TMAO-induced exacerbation in neurodegenerative diseases. Dietary choline is metabolized by gut microbiota to Trimethylamine (TMA), which enters the systemic circulation and is subsequently transported to the liver, where it is oxidized to trimethylamine N-oxide (TMAO) by flavin monooxygenases (FMOs). Elevated circulating TMAO may promote systemic and neuroinflammatory responses through different mechanisms, including the activation of inflammatory signaling pathways and disruption of blood–brain barrier (BBB) integrity. Increased BBB permeability may facilitate infiltration of peripheral immune cells, including neutrophils and other leukocytes, into the central nervous system, further amplifying the production of proinflammatory cytokines and mediators. Sustained neuroinflammation and associated cellular stress may contribute to neuronal dysfunction and apoptosis, thereby potentially accelerating the progression or exacerbation of neurodegenerative diseases. BBB, blood–brain barrier; FMO, flavin monooxygenase; TMA, trimethylamine; TMAO, trimethylamine N-oxide.

In contrast, research on ALS suggests that influencing the TMAO pathway could be therapeutically beneficial. Chen et al. conducted a cross-sectional case–control study to evaluate TMAO metabolic pathways in ALS patients. They observed that ALS patients have higher plasma levels of carnitine and betaine, whereas choline, TMAO, and butyrobetaine levels were lower than those of healthy controls. The higher TMAO concentrations were associated with lower upper motor neuron impairment, which suggests an interaction between TMAO metabolism and disease progression [85]. Overall, these findings suggest that the effects of TMAO and its precursors appear to differ across neurological diseases. Therefore, future research is still needed to understand the underlying mechanisms and the exact role of TMAO in the development of these neurological diseases. Figure 2 summarizes the possible underlying mechanisms that link TMAO to neurodegenerative diseases.

### 1.7. TMAO and Association with Disorders of Gut–Brain Interaction

Diseases related to gut–brain interaction, such as Irritable bowel syndrome (IBS) and functional dyspepsia, are characterized by complex interaction between the gastrointestinal tract and the central nervous system. Growing evidence suggests that changes in the gut microbiota and its metabolic products may influence enteric neuronal activity, immunological signaling, visceral sensory pathways, and epithelial integrity [86,87]. Barrier dysfunction is an important factor in the pathology of IBS, as increased intestinal permeability facilitates greater exposure of mucosal immune cells and sensory nerve endings to luminal antigens and microbial products. This can trigger the activation of mast cells and other immune cells and the release of inflammatory mediators [88,89]. Experimental studies have demonstrated that microbiota-associated barrier disruption and inflammatory signaling can induce visceral hypersensitivity, which has been identified clinically as an important feature of IBS and other DGBIs, along with impaired mucosal immunity and microbiome disturbances [89]. TMAO may contribute to this process through its effect on the epithelial intestinal barrier. Previous studies have shown that TMAO exposure is associated with reduced expression of intestinal tight junction proteins, including zonula occludens-1 (ZO-1) and occludin, along with increased inflammatory signaling [90]. Furthermore, this study has shown that TMAO treatment was associated with increased production of proinflammatory mediators through the activation of the NLRP-3 inflammasome pathway. These findings suggest that increased TMAO levels may influence the intestinal microenvironment and potentially increase epithelial permeability. However, these observations are based on laboratory models, and their relevance to patients needs to be established through randomized clinical trials.

TMAO may also interact with other mechanisms involved in DGIBs, such as dysmotility and visceral hypersensitivity. Alteration in intestinal movement can affect microbial composition, nutrient availability and the duration of mucosal exposure to microbial metabolites. Prolonged exposure to microbial metabolites and inflammatory mediators can influence enteric neurons, smooth muscle, and interstitial cells of Cajal (ICCs), thereby creating a bidirectional relationship between microbial metabolism and gastrointestinal function. Chronic mucosal inflammation and impaired barrier function may create abnormal gut–brain signaling [87,88,91]. Although direct evidence linking TMAO to IBS or other DGBI’s remains limited, TMAO should be considered as one important microbial metabolite that may change in response to dietary intake, microbial composition, intestinal motility, and host metabolism. Therefore, future studies that combine TMAO measurement with microbe composition, intestinal permeability, inflammatory markers, gastrointestinal movement, and gut–brain signaling will be crucial for determining whether TMAO contributes directly to DGBI pathophysiology.

### 1.8. TMAO as a Potential Target for Prevention and Treatment of Stroke

Based on the potential role of gut dysbiosis, partly mediated by TMAO, therapeutic strategies that modulate gut microbiota may help prevent and treat stroke. In a prospective study, a traditional Chinese medicine, Sanhuang Xiexin decoction, was used in patients with acute ischemic stroke; TMAO levels were found to be lower than in the control group. The incidence of ischemic events was lower at 3 and 6 months in the treatment group than in the control group [92]. Fecal microbiota transplantation (FMT) into germ-free mice from low versus high TMAO-producing human subjects showed that TMAO production and stroke severity were transmissible traits [93]. Alteration of gut microbiota by calorie restriction in mice led to improved rehabilitation post-ischemic stroke. Bifidobacterium was enriched in a calorie-restricted diet [94]. FMT using microbiomes from younger mice promotes recovery in older mice with ischemic stroke [95]. Microbiota changes were noted after traumatic brain injury (TBI) in rats, which led to elevated levels of TMA in feces and increased levels of TMAO in the ipsilateral brain. FMT ameliorated these changes and subsequently relieved the neurological deficits in TBI [82]. Resveratrol decreases TMAO levels by inhibiting commensal microbial TMA production and bile acid metabolism, which attenuates TMAO-induced atherosclerosis in mice [96].

### 1.9. TMAO as a Potential Target for Treatment of Neurodegenerative Disorders

Preventive measures and drug therapies to reduce the levels of TMAO could offer treatment measures to reduce the risk of neurodegenerative diseases. Dietary interventions like intermittent fasting have been associated with reduced plasma TMAO levels [97]. On the other hand, the Mediterranean diet is rich in TMAO, but it does not alter plasma levels of TMAO in healthy individuals [98]. It is unclear whether lowering dietary levels alone is sufficient to decrease plasma TMAO levels.

Altering gut flora could change plasma TMAO levels. Antibiotics were shown to markedly suppress plasma TMAO, which reappeared after withdrawal [99]. Probiotics were shown to decrease plasma TMAO levels [100]. Inhibition of certain enzymes associated with TMAO synthesis is another target for altering plasma TMAO levels. Inhibition of the FMO3 enzyme, which converts TMA to TMAO, could also decrease the levels of TMAO. TMA lyase is another potential enzyme to suppress plasma TMAO levels. Several drugs are currently under study that could lower plasma TMAO levels. Several Chinese medications have been shown to lower TMAO levels in animal studies [101]. More studies are needed to establish any role of these drugs in the prevention of neurodegenerative disorders. A certain choline analog, 3,3-dimethyl-1-butanol (DMB), inhibits TMAO production by inhibiting TMA [102]. A meta-analysis evaluating the role of prebiotics and phytochemicals on gut microbiota and TMAO showed significant alterations in the alpha- and beta-diversity of gut microbiota. Probiotics like *Bifidobacterium* and *Akkermansia* were effective in reducing TMAO levels [103]. Taurisol supplement obtained from Grape Pomace Polyphenolic Extract was found to decrease the blood levels of TMAO in a double-blind, randomized, single-center, placebo-controlled, crossover study [103].

## 2. Roadblocks in Targeting TMAO

Although the growing evidence supports TMAO as a potential therapeutic target for neurodegenerative diseases, several limitations must be addressed. First, most of the clinical studies were observational, making it difficult to establish a causal relationship between serum TMAO levels and the progression of neurodegenerative diseases. Furthermore, TMAO has important physiological functions as an osmolyte and chemical chaperone, and its effects depend mainly on concentration, disease context, and duration of exposure [104]. Therefore, eliminating TMAO could interfere with its physiological function. Additionally, circulating TMAO levels can be affected by multiple factors, such as food ingested, gut microbiota composition, liver metabolism, and renal function [105]. This makes it difficult to consistently evaluate TMAO levels and maintain a specific therapeutic range. Moreover, strategies to decrease TMAO levels by modifying the gut microbiota could disrupt other beneficial microbiota populations. Finally, although TMAO has been detected in the central nervous system, the proportion locally produced in the brain versus translocated from the systemic circulation is not fully understood. Therefore, it is unclear whether decreasing circulating TMAO will effectively decrease TMAO levels in the brain. Notably, future studies should focus on identifying the underlying pathological mechanisms of TMAO in neurodegenerative diseases and investigating a therapeutic agent that can limit its harmful effects without disrupting the essential functions of the gut microbiota and host metabolism.

## 3. Conclusions

In conclusion, strong evidence supports the role of TMAO in both neurovascular diseases and neurodegenerative disorders. Future studies are required to understand the definitive role of TMAO in neurodegenerative diseases. Studies are also needed to evaluate the effects of reducing TMAO on the course of neurodegenerative and neurovascular diseases. Additionally, future research should focus on developing drugs to reverse these effects by modulating gut microbiota or lowering TMAO levels. Furthermore, studies are needed to investigate the role, if any, of probiotics that can alter the microbiota composition to promote a decrease in the production of TMA or lower the levels of TMAO in patients at risk for ischemic stroke or neurodegenerative diseases, as well as following the stroke to improve outcomes after the ischemic event.

## Data Availability

No new data were created or analyzed in this study. Data sharing is not applicable to this article.
